# 2490 Research navigation services and onboarding: Succeeding in the research environment

**DOI:** 10.1017/cts.2018.223

**Published:** 2018-11-21

**Authors:** Rebecca Namenek Brouwer, Geeta Swamy

**Affiliations:** Duke University

## Abstract

OBJECTIVES/SPECIFIC AIMS: Describe (1) the components of the research navigation service and consultation/onboarding program, (2) use and adoption of the services, and (3) the overall satisfaction from the research community. METHODS/STUDY POPULATION: Duke offers 2 programs to support researchers: Research Navigation and Researcher Onboarding. The services aim to connect researchers to resources, offices, funding opportunities, and other collaborators. The general Research Navigation Service is an on-demand “hotline,” where navigators answer questions from researchers across the institution, helping them understand processes, best practices, and how to locate resources or potential collaborators. Navigators can be reached via the myRESEARCHhome portal, email, or by phone. The researcher onboarding program is a free 1:1 consultative service, focused on the researcher’s individual portfolio, stage of career, and immediate plans in the research arena. The goal is to equip researchers “from the start” to be successful. Researchers are identified via the new faculty hire list, or by referral. Both services are provided by the myRESEARCHnavigators team, who are trained in a variety of research areas, from basic to clinical to social sciences, and are familiar with Duke. RESULTS/ANTICIPATED RESULTS: Use of both services has increased substantially over the year. Of the almost 200 faculty members hired into the School of Medicine in 2017, ~75% have taken part in the onboarding program, and 91% have rated the service as 5-stars. The content of the sessions will be described. The Research Navigation service has fielded hundreds of calls since its inception, with topics including Equipment and Facilities (55 requests), Study start up (44 requests), Innovation and Technology (15 requests), and Regulation and Policy (25 requests). Categorization of requests, users of the services, and other information about the programs will be described. DISCUSSION/SIGNIFICANCE OF IMPACT: The navigation and onboarding services are proving to be a successful way to increase efficiency and understanding of processes and resources across the institution. Feedback from the users, coupled with high referral rates to the programs, suggests that the program is helping researchers feel better equipped with regard to their research planning, conduct, and analysis.

